# Canine Natural Killer Cell-Derived Exosomes Exhibit Antitumor Activity in a Mouse Model of Canine Mammary Tumor

**DOI:** 10.1155/2021/6690704

**Published:** 2021-09-04

**Authors:** Jienny Lee, Se-A Lee, Na-Yeon Gu, So Yeon Jeong, Jeong Su Byeon, Da-Un Jeong, In-Ohk Ouh, Yoon-Hee Lee, Bang-Hun Hyun

**Affiliations:** ^1^Viral Disease Research Division, Animal and Plant Quarantine Agency, 177 Hyeoksin 8-ro, Gimcheon, Gyeongsangbuk-do 39660, Republic of Korea; ^2^Division of Regenerative Medicine Safety Control, Department of Chronic Disease Convergence Research, Korea National Institute of Health, Korea Disease Control and Prevention Agency, 202 Osongsaengmyeong 2-ro, Cheongju, Chungcheongbuk-do 28159, Republic of Korea

## Abstract

Natural killer (NK) cells are key immune cells engaged in fighting infection and malignant transformation. In this study, we found that canine NK cell-derived exosomes (NK-exosomes) separated from activated cytotoxic NK cell supernatants express specific markers including CD63, CD81, Alix, HSP70, TSG101, Perforin 1, and Granzyme B. We examined the antitumor effects of NK-exosomes in an experimental murine mammary tumor model using REM134 canine mammary carcinoma cell line. We observed changes in tumor size, tumor initiation, progression, and recurrence-related markers in the control, tumor group, and NK-exosome-treated tumor group. We found that the tumor size in the NK-exosome-treated tumor group decreased compared with that of the tumor group in the REM134-driven tumorigenic mouse model. We observed significant changes including the expression of tumorigenesis-related markers, such as B cell-specific Moloney murine leukemia virus insertion site 1 (Bmi-1), vascular endothelial growth factor (VEGF), matrix metallopeptidase-3 (MMP-3), interleukin-1*β* (IL-1*β*), IL-6, tumor necrosis factor-*α* (TNF-*α*), multidrug resistance protein (MDR), tumor suppressor protein p53 (p53), proliferating cell nuclear antigen (PCNA), and the apoptotic markers, B cell lymphoma-2 associated X (Bax) and B cell lymphoma-extra large (Bcl-xL) belonging to the Bcl-2 family, in the tumor group compared with those in the control group. The expression of CD133, a potent cancer stem cell marker, was significantly higher than that of the control. By contrast, the NK-exosome-treated tumor group exhibited a significant reduction in Bmi-1, MMP-3, IL-1*β*, IL-6, TNF-*α*, Bax, Bcl-xL, and PCNA expression compared with that in the tumor group. Furthermore, the expression of CD133, which mediates tumorigenesis, was significantly decreased in the NK-exosome-treated tumor group compared with that in the tumor group. These findings indicate that canine NK-exosomes represent a promising therapeutic tool against canine solid tumors, including mammary carcinoma.

## 1. Introduction

Natural killer (NK) cells play an important role in the immune response against various pathogens. NK cells are innate lymphocytes that provide a rapid defense against tumors and viral infections resulting in pathogen elimination or limited viral spread [1]. The rapid response is primarily attributed to the expression of multiple germline-encoded activating receptors, among which natural cytotoxic receptors and NKG2D are the most important for the recognition and killing of infected cells [2]. NK cells have been characterized in various mammals; however, canine NK cells have yet to be fully characterized.

In 2013, Randy Schekman, James Rothman, and Thomas C. Südhof were awarded the Nobel Prize in Physiology or Medicine for their discovery of the regulatory mechanisms underlying the main intracellular vesicle transport system. Further studies have suggested that exosomes play an important role in several physiological and pathological processes [3], and recently, exosomes have emerged as a novel therapeutic platform [4]. Exosomes are membrane vesicles with an approximate diameter range of 50-200 nm and are formed by cellular endocytosis during intercellular communication [5]. Various research groups have reported that mesenchymal stem cell- (MSC-) derived exosomes play a role in renal disease, myocardial infarction, stroke, brain injury, and other pathologies [6–9]. Fan et al. reported that human fetal liver MSC-derived exosomes also impair the NK cell function [10].

Recently, immune cell-derived exosomes have emerged as novel therapeutics [11]. They regulate cancer progression and metastasis and play a mediating role between innate and adaptive immunity. Chimeric antigen receptor- (CAR-) based T cell adoptive immunotherapy is a distinct and promising cancer therapy. Tang et al. demonstrated the potential therapeutic efficacy of CAR-T cell-derived exosomes as a cell-free modality for cancer therapy [12]. As the sentinel antigen-presenting cells of the immune system, dendritic cells (DCs) play a central role in initiating antigen-specific immunity and tolerance. Pitt et al. reported the potential functional differences between DC- and DC-derived exosome-based cancer therapies [13]. Tian and Li reported the promise and challenges of DC-derived exosomes for cancer immunotherapy [14]. Fais reported that human NK cell-derived exosomes (NK-exosomes) release NK cell-related markers and cytotoxic granules [15]. Recent studies have confirmed that NK-exosomes exert effects on hepatocellular carcinoma, melanoma, lung cancer, multiple myeloma, and neuroblastoma [16].

Canine mammary tumors are one of the most commonly diagnosed in female dogs, and approximately 50% of these are malignant [17]. The primary aim of this study was to investigate whether canine NK-exosomes respond to tumor cell-driven solid tumorigenesis. REM134, a canine mammary carcinoma cell line, is an excellent in vitro model for the analysis of cancer-associated genes and the study of cancer biology and progression [18].

We sought to investigate various immune cell-based treatments using exosomes in the field of veterinary medicine on the basis of research advances in human medicine. Also, we investigated whether canine NK-exosomes exhibit antitumor effects against REM134-driven canine mammary tumorigenesis. We isolated exosomes from activated canine NK cells, which express cytotoxic NK cell receptors, and evaluated their effects in a REM134-driven canine mammary tumor model. To our knowledge, this study is the first to show the antitumor effects of canine NK-exosomes in a canine mammary tumor model.

## 2. Materials and Methods

### 2.1. Isolation and Activation of Canine NK Cells

Canine NK cells were isolated using previously described methods [19]. Peripheral blood mononuclear cells (PBMCs) were isolated from a beagle dog (Genia Inc., Korea). Subsequently, CD5 negative (CD5^lo^) cells were isolated by immunomagnetic separation and cultured in a cell culture flask at 37°C in a 5% CO_2_ incubator supplemented with 500 U/mL human IL-2, 10 ng/mL human IL-15, and 5 ng/mL canine IL-21 (all from R&D System, Minneapolis, USA). After 21 days, cell surface markers were analyzed via FACS flow cytometry of activated CD5^lo^ cells. The activated canine NK cell markers were identified by labeling, CD5^lo^ cells (1 × 10^5^) with antibodies against surface markers including mouse anti-dog CD3-FITC (clone CA17.2A12, Bio-Rad, Hercules, USA), rat anti-dog CD4-FITC (clone YKIX302.9, Bio-Rad), rat anti-dog CD5-PE (clone YKIX322.3, Bio-Rad), mouse anti-dog CD21-APC (clone CA2.1D6, Bio-Rad), rat anti-dog CD45-APC (clone YKIX716.13, Bio-Rad), and rat anti-dog major histocompatibility complex (MHC)-II-FITC (clone YKIX334.2, eBioscience, San Diego, USA) for 1 hr. The labeled cells were washed twice with phosphate buffered saline and analyzed using a FACSCalibur™ flow cytometer (Becton Dickinson, USA) with Cell Quest Pro software (Becton Dickinson) for data analysis.

### 2.2. Cytotoxicity of Canine NK Cells

Cytotoxicity of canine NK cells was determined using a CytoTox 96 nonradioactive cytotoxicity assay (Promega, Madison, USA) according to the manufacturers' instructions. Activated CD5^lo^ cells representing effector cells (E) and REM134 serving as target cells (T) were cocultured at various E:T ratios (25 : 1, 12.5 : 1, 6.25 : 1, 3.13 : 1, and 1.56 : 1). After incubating for 4 hr, the culture supernatants were collected and analyzed to measure the levels of lactate dehydrogenase (LDH), a stable cytosolic enzyme, which is released during cell lysis.

### 2.3. Enzyme-Linked Immunosorbent Assay (ELISA) of Interferon-Gamma (IFN-*γ*)

The level of IFN-*γ* was determined using a canine IFN-gamma DuoSet ELISA kit (R&D System) following the manufacturers' instructions. CD5^lo^ cells (2 × 10^6^) were cocultured with target cells (REM134, 2 × 10^5^) at a 10 : 1 E:T ratio in a 6-well plate. After 24 hr of coculture, the cell-free culture supernatants were harvested and analyzed for IFN-*γ* production. REM134 and CD5^lo^ cells cultured alone for 24 hr were used as the control. The optical density (OD) of each standard, sample, and control was determined at 450 nm on basis of the average OD of duplicates. The OD value was subtracted from the mean blank control to construct a standard curve. The sample concentration was determined using the corresponding mean absorbance from the standard curve.

### 2.4. Isolation and Characterization of NK-Exosomes

Canine CD5^lo^ cells were activated with IL-2, IL-15, and IL-21 for 21 days. The cell culture medium with exosome-depleted fetal bovine serum was replaced, and the culture supernatant was harvested. The supernatant was centrifuged to remove cells and debris and then filtered through a 0.22 *μ*m filter. The filtrate was centrifuged at 100,000 g for 90 min at 4°C via ultracentrifugation. Western blot analysis was performed to measure CD63 (clone H5C6, Novus, Littleton, USA), CD81 (catalog number TA343281, Origene, Rockville, USA), Alix (clone 3A9, Cell signaling technologies, Danvers, USA), HSP70 (clone C92F3A-5, Thermo Fisher, Rockford, USA), TSG101 (catalog number orb11527, Biorbyt, Cambridge, UK), Perforin 1 (clone A-2, Santa Cruz, Dallas, USA), and Granzyme B (clone 496B, Thermo Fisher) in the samples. Exosomes typically measure 40-200 nm in diameter and were characterized via nanoparticle tracking analysis (NTA, Malvern Instruments, Worcestershire, UK).

### 2.5. In Vitro Sphere Formation and Drug Resistance Assay

REM134 cells (2 × 10^5^ cells/well in 1.5 mL of medium) were layered onto ultralow attachment 6-well plates. Spheres were grown in serum-free DMEM/F12 medium (Gibco, Waltham, USA), 10 ng/mL human recombinant basic fibroblast growth factor (Invitrogen, Carlsbad, USA), 10 ng/mL mouse recombinant EGF (Invitrogen), 20 *μ*g/mL insulin (Sigma, St. Luis, USA), 20 nM progesterone (Sigma), 100 *μ*M putrescine chloride (Sigma), 30 nM sodium selenite (Sigma), 25 *μ*g/mL transferrin (Sigma), 0.5% methylcellulose (R&D System), and 1× B-27 supplement (Invitrogen) and allowed to grow for 7-10 days. Spheres were counted and then harvested for protein extraction or split into a second generation of spheres, followed by lysis for protein extraction [20].

### 2.6. Animals

BALB/c nude mice used in these studies were purchased from the Orient Company (Seongnam, Korea). The mice were housed at 23 ± 2°C (humidity 50 ± 5%) under a 12/12 hr light/dark cycle. The mice had free access to food and tap water. Animal experiments were approved by the Animal and Plant Quarantine Agency Guide following Institutional Animal Care and Use Committee (IACUC) guidelines (Approval number 2018-410). All of the procedures in this study were performed according to the Animal and Plant Quarantine Agency Guide and IACUC, which are important guidelines for animal research in the United States.

### 2.7. In Vivo Tumor Xenografts

Canine mammary tumors were induced in mice, which were divided into three groups (control group, tumor group, and NK-exosome-treated tumor group). REM134 cells (1 × 10^4^) were xenografted into the mammary fat pad of BALB/c nude mice. The tumor volume was monitored each week after cancer detection compared with that of the normal group. After 2 weeks of tumor induction, NK-exosomes (100 *μ*g per 1 time) were administered into the tumor site and tail vein twice weekly for 6 weeks. Tumor size and body weight of the mice were recorded in the tumor group and the NK-exosome-treated tumor group in the REM134-driven tumorigenic mouse model [21]. At the end of the experiment, we dissected the tumor tissues from the mammary fat pad and weighed them. Tumor volume was calculated by the following formula:

(1) $$\begin{array}{c} \text{Tumor volume }\left(\text{m}{\text{m}}^{3}\right)=1/2\;\left(\text{length}\times \text{widt}{\text{h}}^{2}\right) \end{array}$$

### 2.8. Quantitative Real-Time RT-PCR (qRT-PCR)

Total RNA was isolated from the cells and tissues and reverse transcribed into cDNA (5 *μ*L), which was used for PCR analysis. The qRT-PCR analysis was performed in 96-well plates with a LightCycler 480 (Roche Applied Science, Germany) using a SYBR Green I Master kit (Roche Diagnostics, Germany) according to the manufacturer's instructions. The primers used for PCR included CD133, B cell-specific Moloney murine leukemia virus insertion site 1 (Bmi-1), vascular endothelial growth factor (VEGF), matrix metallopeptidase-3 (MMP-3), interleukin-1*β* (IL-1*β*), IL-6, tumor necrosis factor-*α* (TNF-*α*), multidrug resistance protein (MDR), B cell lymphoma-2 associated X (Bax), B cell lymphoma-extra large (Bcl-xL), tumor suppressor protein p53 (p53), proliferating cell nuclear antigen (PCNA), and *β*-actin. The thermocycling program used for amplification was as follows: predenaturation (95°C, 10 min), followed by 45 cycles of denaturation (95°C, 10 sec), annealing (60°C, 10 sec), and elongation (72°C, 10 sec). Melting curve analysis was performed from 60°C to 95°C to evaluate the homogeneity of the qRT-PCR products. The qRT-PCR results were calculated using Ct values. Relative quantification was conducted as previously described using *β*-actin as a reference gene. The 2^−*Δ*CT^ method described by Livak and Schmittgen was applied to normalize the gene expression values [22].  Table 1 lists the primer sequences and their respective annealing temperatures.

### 2.9. Statistical Analysis

The relative expression of specific marker genes was analyzed using a one-way analysis of variance, and the differences between the two methods were compared using Student's *t*-test (SigmaPlot 12.0; Systat Software Inc., Erkrath, Germany).

## 3. Results

### 3.1. Characterization of Canine NK Cells

**Figure 1:**
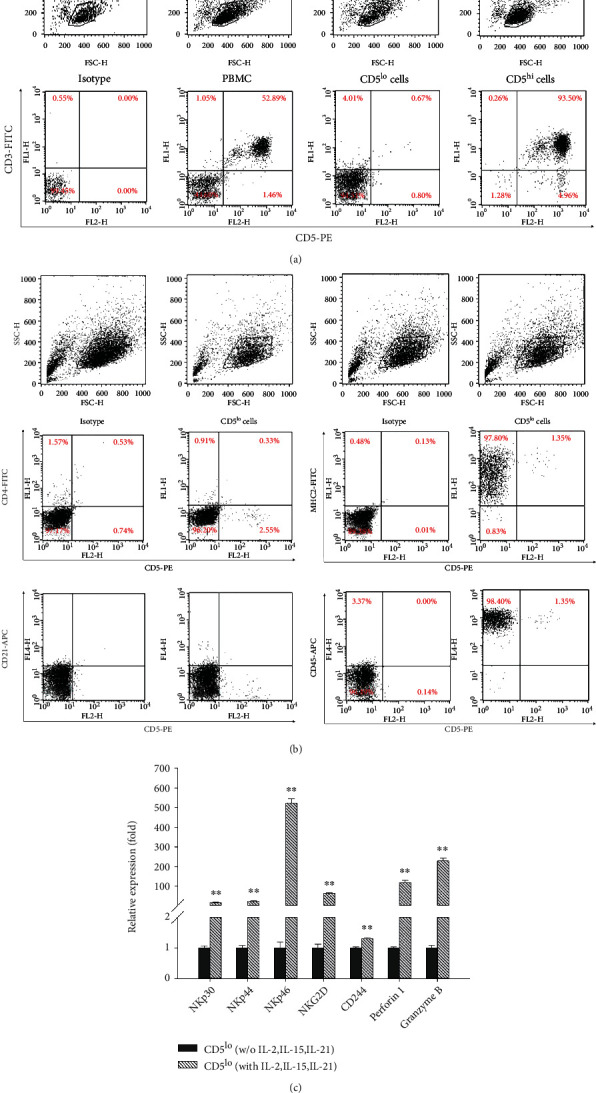
Isolation and characterization of canine NK cells. (a) CD5 negative (CD5^lo^) cells were isolated from canine PBMCs and activated in specific culture supplemented with human IL-2 (500 U/mL), IL-15 (10 ng/mL), and canine IL-21 (5 ng/mL) for 21 days. (b) Cell surface markers were analyzed by FACS flow cytometric analysis at activated CD5^lo^ cells. (c) To determine whether CD5^lo^ cells develop into cytotoxic NK cells, the expression of activated NK cell markers (NKp30, NKp44, NKp46, NKG2D, CD244, Perforin 1, and Granzyme B) in activated canine CD5^lo^ cells was analyzed by quantitative RT-PCR. *β*-Actin was used as a reference gene. ^∗^*P* < 0.05, ^∗∗^*P* < 0.01, ^∗∗∗^*P* < 0.001.

NK cells are a subset of large granular lymphocytes characterized as non-T and non-B cells. Canine NK cells were characterized by isolating CD5^lo^ cells from canine whole blood-derived PBMCs via immunomagnetic separation (Figure 1(a)). The cells were activated under specific culture conditions supplemented with IL-2, IL-15, and IL-21. After 21 days, cell surface markers were analyzed via FACS flow cytometry of activated CD5^lo^ cells. The flow cytometric analysis of activated CD5^lo^ cells indicated that they were positive for CD45 and MHC-II but negative for CD3, CD4, CD5, and CD21 (Figure 1(b)).

It is well known that canine NK cells express fewer cell surface CD5 molecules [23]. Although activated CD5^lo^ cells are not non-T and non-B cells, it is necessary to determine whether CD5^lo^ cells can develop into cytotoxic NK cells against target cells. Thus, we also found that activated canine CD5^lo^ cells express high levels of NK cell-related receptors (NKp30, NKp44, NKp46, NKG2D, and CD244) as well as Perforin 1 and Granzyme B, which are found in NK cell granules via qRT-PCR (Figure 1(c)).

### 3.2. Cytotoxicity of Canine NK Cells

**Figure 2:**
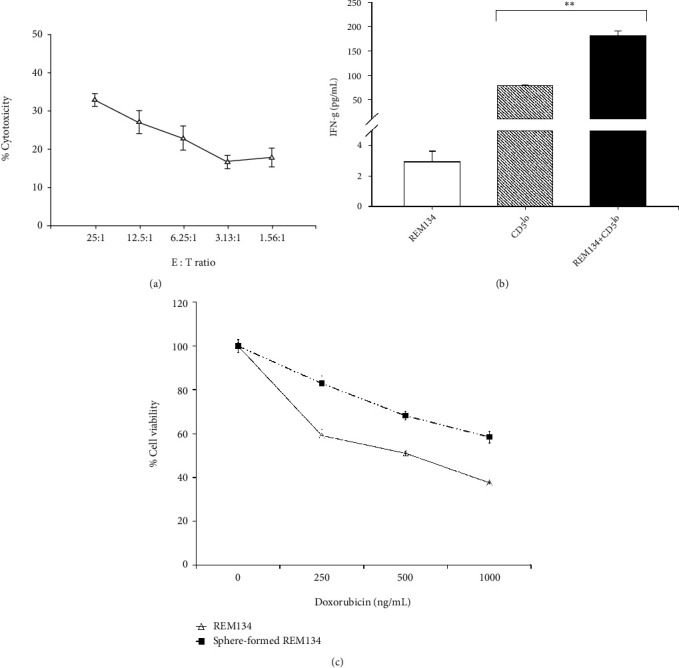
Cytotoxicity of canine NK cells. (a) Activated CD5^lo^ cells as effector cells (*E*) and REM134 as target cells (*T*) were cocultured at the E:T ratios of 25 : 1, 12.5 : 1, 6.25 : 1, 3.13 : 1, and 1.56 : 1. The culture supernatants were collected and analyzed to measure the release of lactate dehydrogenase (LDH) upon cell lysis. (b) Analysis of activated CD5^lo^ cells synthesizing IFN-*γ* against REM134. ^∗^*P* < 0.05, ^∗∗^*P* < 0.01, ^∗∗∗^*P* < 0.001. (c) The sphere-forming assay showed that REM134 generates a malignant cell phenotype.

We investigated whether activated CD5^lo^ cells exhibit cytotoxic activity against REM134 canine mammary carcinoma cells. Activated CD5^lo^ cells as effector cells (E) and REM134 as target cells (T) were cocultured at various E:T ratios (25 : 1, 12.5 : 1, 6.25 : 1, 3.13 : 1, and 1.56 : 1). The culture supernatants were analyzed for LDH, which is released upon cell lysis. Consequently, when activated CD5^lo^ cells were cocultured with REM134, the production of LDH was increased and depend upon the E:T ratio compared with the control (target cells alone). Specifically, activated CD5^lo^ cells (32.88% ± 1.65% at an E:T ratio of 25 : 1) showed a significant NK cytotoxic effect (Figure 2(a)).

The cytotoxic activity of NK cells was validated using IFN-*γ* production and effector-mediated target cell assays. Significant IFN-*γ* production typically indicates NK cell activation [24]. We also investigated whether activated CD5^lo^ cells secrete IFN-*γ* against REM134. Consequently, significant IFN-*γ* release was detected in activated CD5^lo^ cells cocultured with REM134 (Figure 2(b)), suggesting that activated canine CD5^lo^ cells exhibit NK cell characteristics.

One of the most important properties of cancer stem cells (CSCs) is tumorigenesis. It is known that single-cell-derived sphere formation facilitates CSC identification and tumor studies. We found that REM134 induces a malignant type of cell and confirmed the impact of REM134 on the tumorigenic cell phenotype in a sphere-forming assay. The sphere-formed REM134 exhibited the significantly increased expression of CD133 when compared with the control. Next, we analyzed the drug resistance phenotype of sphere-formed REM134 using doxorubicin, which is currently the most effective chemotherapeutic drug used to treat breast cancer [25]. The results indicated that single cell-derived sphere-formed REM134 is significantly resistant to doxorubicin in comparison with the adherent type of REM134. REM134 may exhibit CSC properties upon tumor formation as sphere-formed cells in the three-dimensional environment of the body (Figure 2(c)).

### 3.3. Characterization of Canine NK-Exosomes

**Figure 3:**
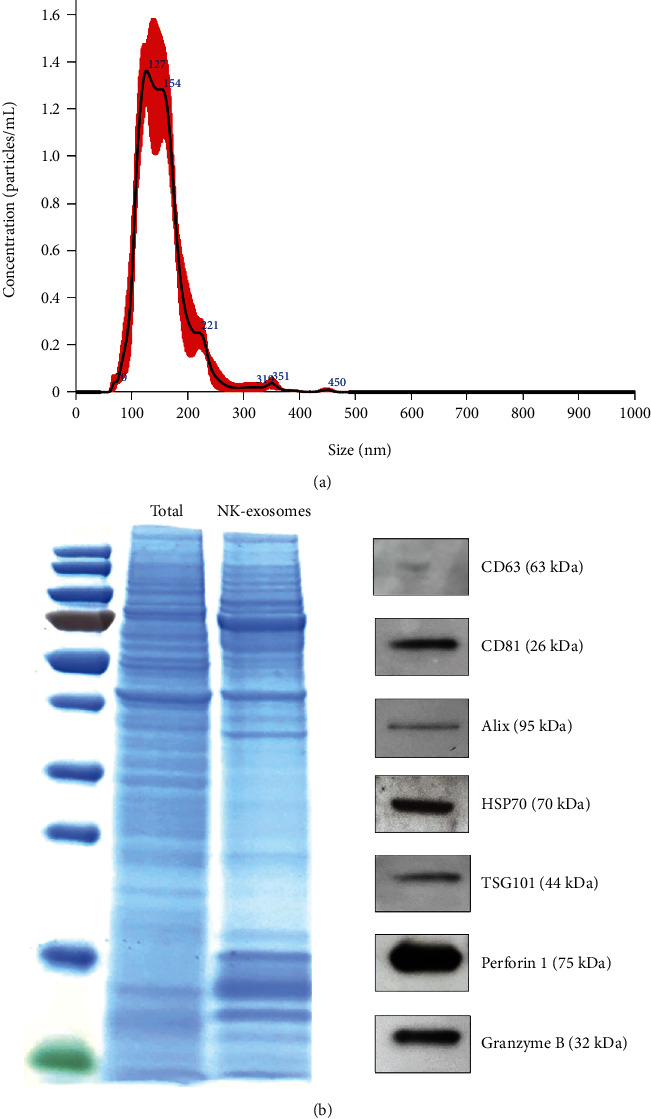
Characterization of canine NK-exosomes. (a) Using nanoparticle tracking analysis, the size of NK cell-derived exosomes (NK-exosomes) was analyzed. (b) Specific markers (CD63, CD81, Alix, HSP70, TSG101, Perforin 1, and Granzyme B) were analyzed in NK-exosomes using western blot analysis.

Next, we isolated and characterized NK-exosomes from cultured supernatants via ultracentrifugation. The isolated NK-exosomes were approximately 136.6 ± 9.4 nm in diameter based on NTA (Figure 3(a)). We also confirmed the expression of specific exosome markers (CD63, CD81, Alix, HSP70, and TSG101) and NK cell markers (Perforin 1 and Granzyme B) in the activated NK-exosomes via western blot analysis (Figure 3(b)). These results suggest that activated canine NK-exosomes represent the properties of NK cells.

### 3.4. Effects of Canine NK-Exosomes on Tumor Progression

**Figure 4:**
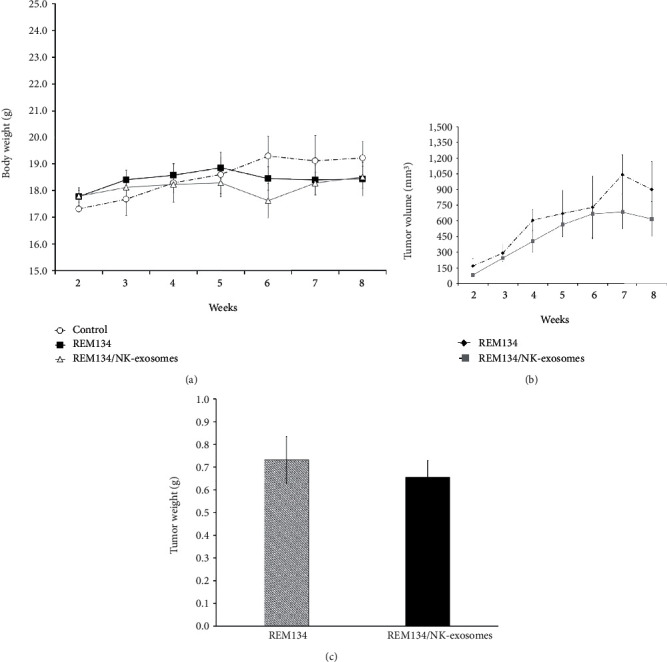
Effects of canine NK-exosomes on tumor progression in a mammary tumor model (a, b) REM134 cells (1 × 10^4^) induced tumor initiation and obvious tumor formation and progression in BALB/c nude mice. After 2 weeks, xenograft mice carrying REM134 were once intratumorally and twice intravenously injected with NK-exosomes each week. Body weight and tumor size of mice were monitored each week until 8 weeks. (c) Tumor tissues from the mammary fat pad were dissected and weighed at 8 weeks.

We established a REM134-driven tumor initiation model and observed tumor formation and progression in BALB/c nude mice. Two weeks after tumor initiation, xenograft mice with REM134 tumors were injected intratumorally once and intravenously twice with NK-exosomes twice each week. Body weight and tumor size were monitored each week until 8 weeks. Significant differences were detected within 4 weeks in only the REM134-driven tumor group in comparison with the tumor group treated with NK-exosomes. Inhibition of tumor growth was observed in the tumor group treated with NK-exosomes but not in the untreated tumor group (Figures 4(a) and 4(b)). At the end of the experiment, we dissected tumor tissues from the mammary fat pad for weight analysis. Tumor weights were reduced in the tumor group treated with NK-exosomes compared with untreated tumor group (Figure 4(c)). These results suggest that activated canine NK-exosomes exhibit antitumor effects.

### 3.5. Effects of Canine NK-Exosomes on the Expression of CSC Markers

**Figure 5:**
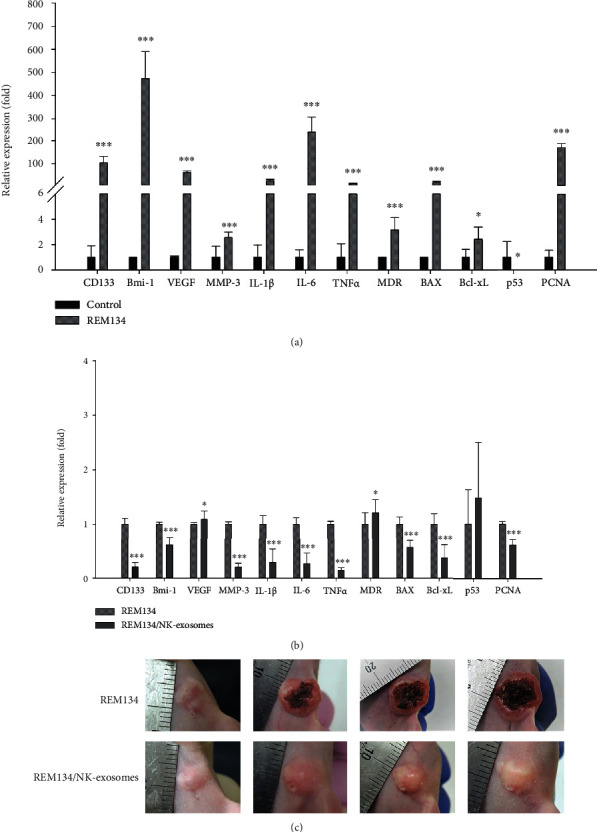
Effects of canine NK-exosomes on the expression of cancer stem cell markers. (a, b) To examine whether activated NK-exosomes influence the expression of CSC-related markers (CD133, Bmi-1, VEGF, MMP-3, IL-1*β*, IL-6, TNF-*α*, MDR, Bax, Bcl-xL, p53, and PCNA), the expression level of tumorigenesis-related marker was examined in control, untreated tumor, and NK-exosome-treated tumor groups. ^∗^*P* < 0.05, ^∗∗^*P* < 0.01, ^∗∗∗^*P* < 0.001. (c) We monitored the tumor volume of the mice in the tumor group (REM134, top) and NK-exosome-treated tumor group (REM134/NK-exosomes, bottom) in the REM134-driven tumorigenic mouse model. Representative images are shown (2, 4, 6, and 8 weeks after tumor induction).

Mounting evidence suggests that CSCs promote tumor progression, metastasis, and drug resistance [26]. Thus, we investigated whether activated NK-exosomes regulate the expression of CSC-related markers on NK-exosomes during antitumor therapy. The results revealed significant changes including the increased expression of tumorigenesis-related markers, such as CD133, Bmi-1, VEGF, MMP-3, IL-1*β*, IL-6, TNF-*α*, and MDR, as well as the apoptotic markers, Bax and Bcl-xL, in the REM134-driven tumor group. Among them, the expression of CD133, a potent CSC marker, was significantly higher than that of the control. We also found that the expression of PCNA was increased in the REM134-driven tumor group, whereas the expression of p53, a tumor suppressor gene, was significantly reduced (Figure 5(a)). However, the expression of HER-2, which is overexpressed in human breast cancer patients, was not significantly elevated in the canine mammary carcinoma mouse model (data not shown).

The NK-exosome-treated tumor group showed a meaningful reduction in the expression of Bmi-1, MMP-3, IL-1*β*, IL-6, TNF-*α*, Bax, and Bcl-xL compared with the tumor group. Furthermore, we confirmed that the expression of CD133, which has a functional role in tumorigenesis, was decreased in the NK-exosome-treated tumor group compared with the control. Additionally, the NK-exosome-treated tumor group showed the reduced expression of PCNA, whereas the expression of p53 was slightly increased compared with that of the tumor group. This suggests that activated canine NK-exosomes exert antitumor effects by downregulating CSC-related markers and enhancing the tumor suppressive function of p53 in this canine mammary tumor murine model (Figures 5(b) and 5(c)).

## 4. Discussion

Canine tumors are invaluable models for the study of human cancers [27, 28]. Among the canine tumors, canine mammary tumors are the most frequent neoplasm in female dogs, and more than 50% are malignant [29–32]. In recent years, several studies have advanced the understanding of the genetic basis for canine mammary tumors [33–36]. Kim et al. reported cross-species oncogenic signatures for breast cancer in canine mammary tumors. They found molecular and histological discrepancies between canine mammary tumors and human breast cancers, despite their similarities [37]. For example, the amplification of HER2, a member of the human epidermal growth factor receptor family, has been shown to play an important role in the development and progression of certain aggressive types of human breast cancer. The HER2 amplification occurs in 20-25% of human breast cancers and is associated with a high rate of relapse and poor prognosis [38]. Trastuzumab (Herceptin®) is a monoclonal antibody that inhibits downstream signal transduction by targeting the extracellular domain of the HER2 receptor [39]. However, the efficiency of Herceptin® is approximately 26%, even in HER2-overexpressing human breast cancer patients [40]. Thus, the clinical benefit and the specific strategy applied against a target gene may not be straightforward in canine mammary tumors.

Recent studies have reported subpopulations of cells known as CSCs within tumors that are responsible for tumor initiation, expansion, metastasis and recurrence after therapy. Tumors contain a large number of cancer cells along with a small population of CSCs [41]. CSCs are resistant to multiple cancer therapies, including chemotherapy and radiation therapy [42]. A single cell-derived sphere formation assay can be used to identify CSCs and their drug resistance mechanisms [43–45]. Michishita et al. reported that sphere-formed cells derived from canine mammary adenocarcinoma cell lines exhibit characteristics of CSCs [43]. In this study, we found that REM134 generates cellular spheres and confirmed their impact on the tumorigenic cell phenotype of REM134. We confirmed that sphere-formed REM134 cells exhibit significant doxorubicin resistance compared with adherent-type REM134 cells. Hence, it is expected that REM134 may exhibit properties of CSCs upon tumor formation as sphere-formed cells in the three-dimensional environment of the body.

We focused on reducing the functional potential of CSCs and CSC-related markers to drive tumor initiation, expansion, metastasis, and recurrence. We induced REM134-driven tumor initiation and monitored tumor formation and progression in BALB/c nude mice. Expectedly, tumor formation and growth rate were accelerated by the entry of a small number of cells into the body within 2 weeks, which represents a very serious phase in humans. Surprisingly, the tumor growth was inhibited in mice treated with NK-exosomes compared with untreated mice. These results suggest that activated canine NK-exosomes exert antitumor effects. CSC-targeted therapy is an interesting area of cancer research. Evidence increasingly suggests the presence of CSCs in various solid tumors, with a potential role in tumor initiation, progression, and recurrence [43]. Thus, the suppression of CSCs is an important strategy for the development of novel therapeutic agents and for the elucidation of the molecular and cellular mechanisms underlying tumorigenesis in veterinary research.

The immune system exploits highly destructive mechanisms. NK cells are a type of immune cell that applies activating receptors, such as NKG2D, to recognize and eliminate infected and transformed cells that upregulate the ligands for these receptors. Six to eight different NKG2D ligands are poorly expressed by normal cells but are upregulated in cancer cells [46]. Canine NK cells exhibit lower levels of CD5 molecules [23]. We confirmed that activated canine CD5^lo^ cells express high levels of NKG2D and NK cell-related activating receptors. We also found that activated canine CD5^lo^ cells exhibit cytotoxic NK cell properties against target cells through LDH and IFN-*γ* production. This study is the first of its kind to examine the antitumor effect of NK-exosomes in a canine mammary carcinoma murine model. A query of the Clinical Trials website (https://clinicaltrials.gov) using the search term “exosomes” returned more than 100 clinical studies. However, clinical studies investigating human breast cancer using exosomes were not identified until June 2021. We focused on NK-exosomes that decrease the functional potential of CSCs and CSC-related markers that drive tumor initiation, expansion, metastasis, and recurrence. We observed significant changes in tumorigenesis-related markers including CD133, Bmi-1, VEGF, MMP-3, IL-1*β*, IL-6, TNF-*α*, and MDR in the tumor group compared with the control group.

Several CSC markers were also identified. CSCs are a limited subpopulation of cells with stem cell-like properties. CD133 is a transmembrane protein expressed on the surface of hematopoietic stem cells and is widely recognized as a CSC marker. CD133 is also strongly associated with cancer-related signaling pathways and promotes tumor invasion, progression, and migration [47]. Several reports suggest that CD133 is a valuable prognostic marker in melanoma, prostate cancer, and glioma [48–50]. Kim et al. suggested that CD133-expressing breast cancer cells exhibit tumorigenesis, tumor self-renewal, high tumor cell proliferation, and drug resistance properties [51].

Liu et al. reported that the overexpression of Bmi-1, polycomb complex protein, in normal mammary epithelial cells increased sphere formation [52]. Bmi-1 has been shown to regulate the self-renewal of both normal and malignant mammary stem cells [53]. Thus, the suppression of Bmi-1 expression is related to the functional inhibition of CSCs, which form malignant tumor cells. Vascularization is an important characteristic of breast cancer development. Accordingly, the regulation of angiogenic factors has been suggested to play a therapeutic role [31, 54]. Pakravan et al. reported that MSC-derived exosomes regulate the mTOR/HIF-1*α* signaling axis in human breast cancer cells, resulting in a significant reduction of the VEGF expression [55]. Lee et al. also reported that MSC-derived exosomes suppress angiogenesis by downregulating VEGF in murine breast cancer cells [56]. MMPs are zinc-dependent endopeptidases that constitute a family of 24 members in mammals [57]. The overexpression of MMP-3 had been shown to drive the formation of mammary tumors in mice [58].

IL-1*β*, IL-6, and TNF-*α* are major proinflammatory cytokines. Cytokines represent key mediators of inflammation and play an important role in the interaction between inflammation and cancer. Proinflammatory states are associated with carcinogenesis [59]. Cancer is a highly heterogeneous disease, both within a single patient as well as among different patients. Various single and combination therapies have been developed and refined for effective treatment. Recently, the evolution of MDR in cancer represents a major challenge to successful treatment. MDR is a complex process involving multiple noncellular and cellular mechanisms [60]. In this study, we found that compared with the tumor group alone, the NK-exosome-treated tumor group exhibited a significant decrease in the expression of CD133, Bmi-1, MMP-3, IL-1*β*, IL-6, and TNF-*α*.

The Bcl-2 family of proteins is prominently involved in the promotion or inhibition of apoptosis [61]. The proapoptotic multidomain molecules, Bax and Bak, mediate mitochondrial apoptosis, whereas Bcl-xL, Mcl-1, Bcl2a1, and Bcl-w are common prosurvival and antiapoptotic proteins [62]. The balance of proapoptotic and antiapoptotic proteins regulates the fate of cells [63]. The p53 tumor suppressor gene prevents tumor development through the inhibition and elimination of abnormally proliferating cells. It is known that p53 mutations are associated with poor prognosis and tumor aggressiveness [64]. He et al. reported that MSCs inhibit tumor progression and enhance the radiosensitivity of human breast cancer cells. MSC-conditioned medium suppresses the growth of breast cancer cells by regulating stemness genes (Sox2, Oct4, Nanog, and c-Myc) and Stat3 signaling pathway-related genes (cyclin D1, Bcl-xL, and p53) [65]. We also found that in the REM134-driven tumor group, the expression of Bax, BcL-xL, and PCNA was increased, whereas the expression of p53 was decreased. By contrast, the NK-exosome-treated REM134-driven tumor group showed significantly reduced expression of Bax, Bcl-xL, and PCNA, whereas the reduced expression of p53 was similar to that of the tumor group. Therefore, NK-exosomes exert antitumor effects by downregulating CSCs and tumorigenesis-related markers and modulating the apoptotic protein expression. This study provides evidence supporting the role of CSCs in tumorigenesis in a model of canine mammary carcinoma. Further studies are required to elucidate the antitumor mechanism of NK-exosomes in more detail.

## Figures and Tables

**Table 1:** List of primers used for the reverse transcription-polymerase chain reaction.

Gene	Primer sequence (5′-3′)	Annealing temperature (°C)	Accession number
CD133	F-AGC CCT GTT GAA CGT GAA CA	60	KJ654317
	R-GTT GTA GCC ACT GGA GGG AC		

Bmi-1	F-CAC TGT GAA TAA TGA CTT CTT GCA T	60	NM_001287063
	R-AAG TTT ACT TTC CTT TGA TCG GTT T		

VEGF	F-GTA ATG ATG AGG GCC TAG AGT G	60	NM_001003175
	R-TAT GTG CTG GCC TTG ATG AG		

MMP-3	F-GTT GGA GGT GAC AGG GAA GG	60	AY183143
	R-CCA GGG AAG GTG GTG AAG TC		

IL-1*β*	F-GCC AAG ACC TGA ACC ACA GT	60	NM_001037971
	R-TGA CAC GAA ATG CCT CAG AC		

IL-6	F-CCT GGT GAT GGC TAC TGC TT	60	U12234
	R-TTG TTT GCA GAG GTG AGT GG		

TNF-*α*	F-GAG CCG ACG TGC CAA TG	60	Z70046
	R-CAA CCC ATC TGA CGG CAC TA		

MDR	F-GAG GAC TTG AAT GAG AAT GTT CCT	60	[60]
	R-CGG GTA AAG ATC CCT ATA ATC CTT		

Bax	F-CCG TGA GGT CTT CTT CCG AG	60	AB080230.1
	R-TAG AAG AGG GCA ACA ACC CG		

Bcl-xL	F-AAG GCG TTT CAG AGA AAA GGG	60	AB073983.1
	R-TTT TGA ATC ACC ACA CCG GC		

p53	F-TTC CTC CCC GAT GGC TCT TA	60	CFU62133
	R-AGA TGC CAA ACC AGA CCT CG		

PCNA	F-TCC TGC GCA AAA GAT GGA GT	60	XM_534355.4
	R-GAG AGA GCG GAG TGG CTT TT		

NKp30	F-TTG GCT CTG TCA CGT GGT AC	60	DQ003484
	R-CAG TGT CCC ATT CCC TGT CC		

NKp44	F-ATC GAG TGG CAG GGC AGA CA	60	XM_0846203
	R-TTC CTC CTT CAG ACC AAT CAT GGT		

NKp46	F-CCA GCA GAG CCC AAA ACA AC	60	NM_001284448
	R-CGG GAT GAA CGG AGA GAG TG		

NKG2D	F-ACG AAG GCA AAA GAG AAA GCC	60	XM_849013
	R-TGA TGA TTA TGG CAC CGC AT		

CD244	F-GGA GGA GGC TGG GGT TTT AC	60	XM_022415323
	R-GCG CCA TCT ACT CTC ACT CC		

Perforin 1	F-GAC AGA GCC CGT TGG AAG AA	60	FJ973622
	R-TTG GTG ATC CCC GAG TTG TG		

Granzyme B	F-GGA ACA GGA GAA GAC CCA GC	60	XM_547752
	R-TTG GCC TTC CTC TCA AGC AG		

*β*-Actin	F-GCT ACG TCG CCC TGG ACT TC	60	NM_001003349
	R-GCC CGT CGG GTA GTT CGT AG		

## Data Availability

The datasets used and/or analyzed during the current study are available from the corresponding author on reasonable request.
